# Supplementary figures and images for: Genetic analysis and fine mapping of a qualitative trait locus wpb1 for albino panicle branches in rice
Source: PLoS One. 2019 Sep 26;14(9):e0223228. doi: 10.1371/journal.pone.0223228 (PMC6763196; doi:10.1371/journal.pone.0223228)

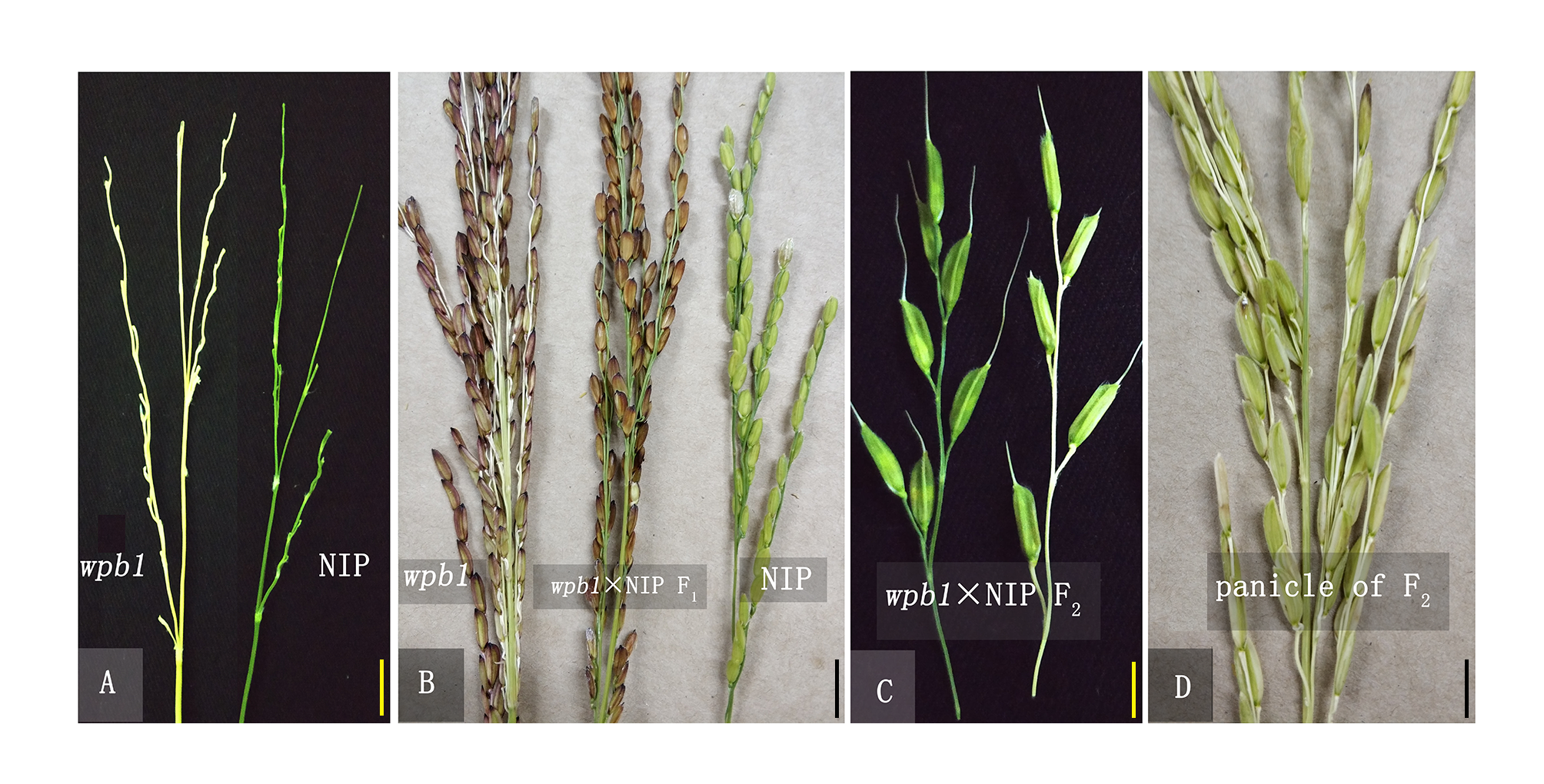

Supplement: S1 Fig — A. The panicle branches of Nipponbare and wpb1. B. The panicle branches of F1 plant. C and D. The panicle branches of F2 plant. Bar = 1 cm. (TIF) [file pone.0223228.s001.tif]

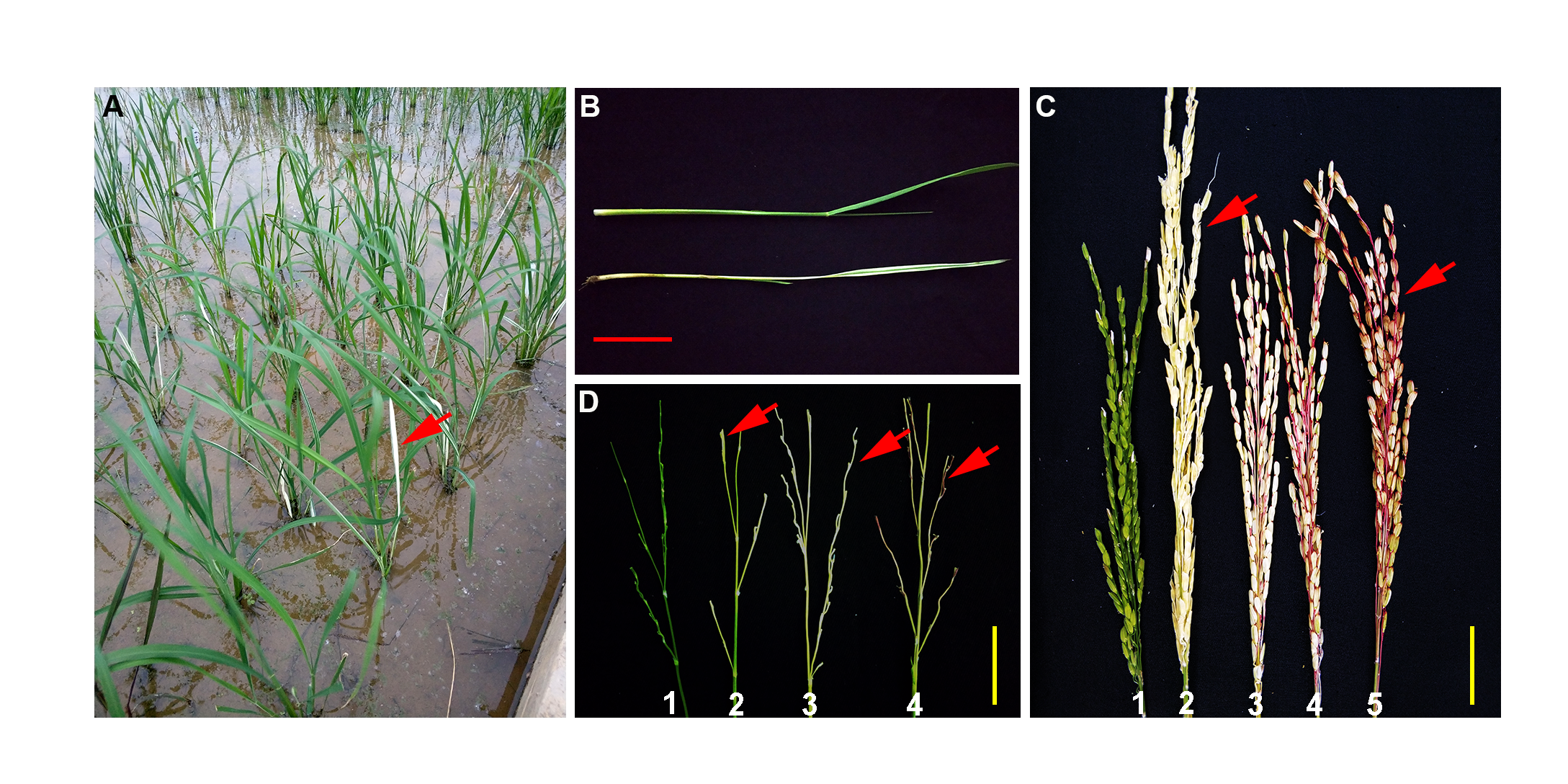

Supplement: S2 Fig — A. wpb1 mutant in the tillering stage. B. The tiller of NIP (top) and wpb1 mutant (bottom). C. The spike phenotype of the wpb1 mutant at different heading temperatures. 1 NIP, 2 heading at 30°C, 3 heading at 25°C, 4 and 5 heading at 18°C. As the temperature decreases, the branches change from white to red. D. Branches phenotype. 1 NIP, 2 heading at 30°C, 3 heading at 25°C, 4 heading at 18°C. Bar, 3 cm (B). 5 cm (C). 8 cm (D). (TIF) [file pone.0223228.s002.tif]

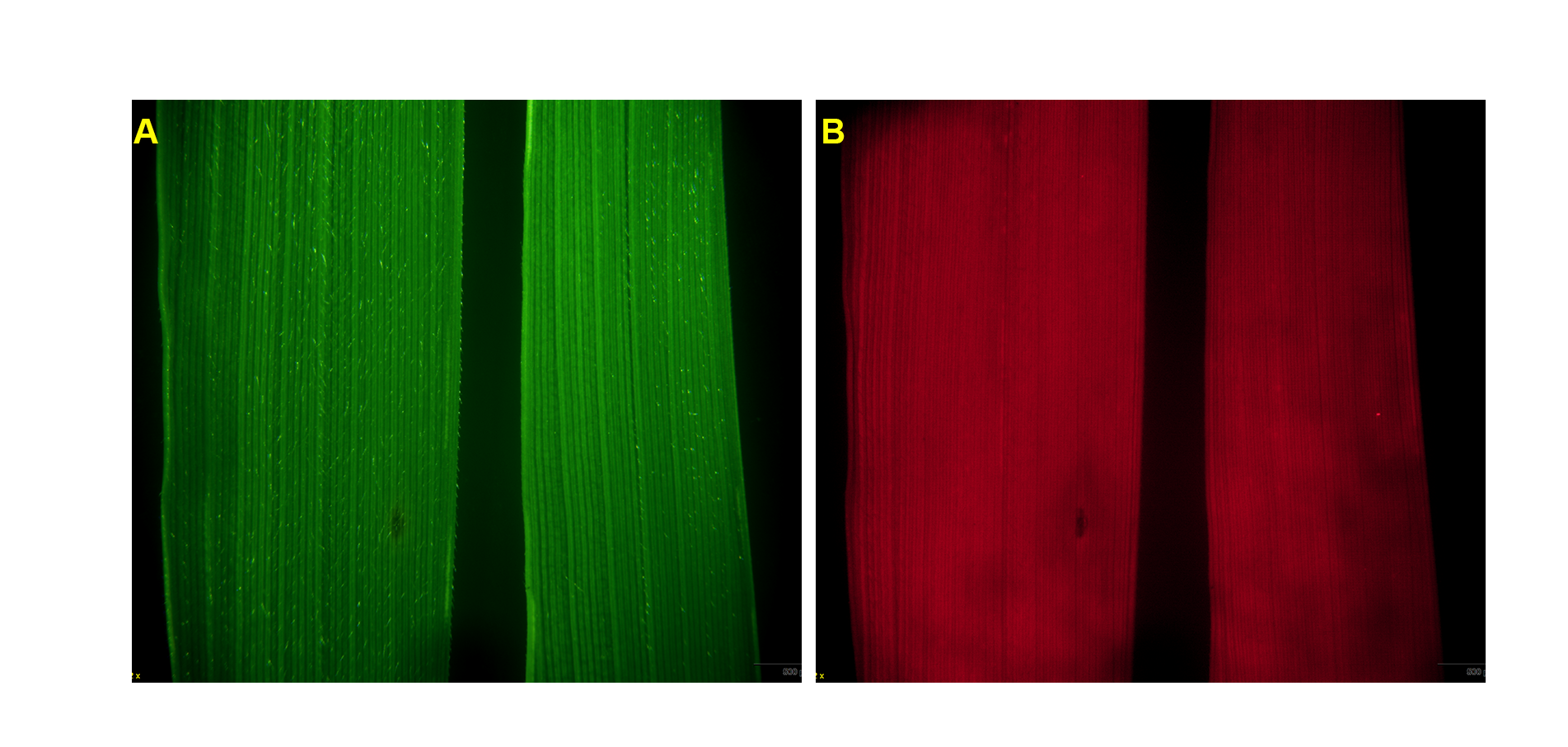

Supplement: S3 Fig — A. bright field. B. RFP field. Chlorophyll can fluoresce in the RFP field. Bright field: Exposure time 300 ms, ISO 200. RFP field: Exposure time 1 s, ISO 1600. (TIF) [file pone.0223228.s003.tif]

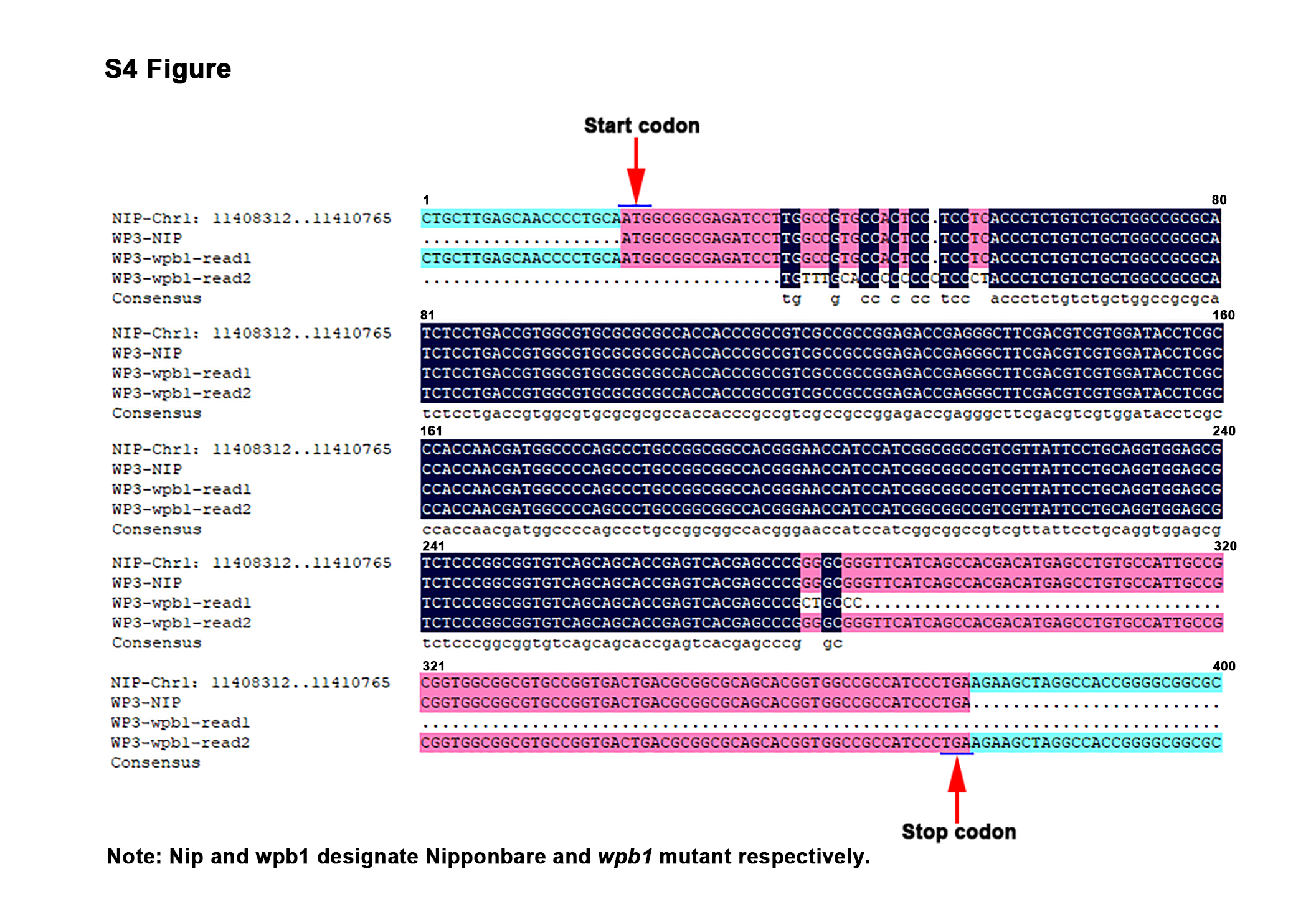

Supplement: S4 Fig — (TIF) [file pone.0223228.s004.tif]
