# Supplementary material for: Genetic analysis and fine mapping of a qualitative trait locus wpb1 for albino panicle branches in rice
Source: PLoS One. 2019 Sep 26;14(9):e0223228. doi: 10.1371/journal.pone.0223228 (PMC6763196; doi:10.1371/journal.pone.0223228)
Supplement: S1 Table — The list of primers of InDel markers for fine mapping obtained from Illumina sequencing. (DOCX) [file pone.0223228.s005.docx]

**S1** **Table. List of fine mapping primers used in this study**

| **Primer Name** | **Sequence 5'-3'** | **Primer Name** | **Sequence 5'-3'** |
| --- | --- | --- | --- |
| M1F | AGCGAGGCTGGTTGATG | M7F | GGAGAAGAAAGCTCACCTGCT |
| M1R | TGAGCCTAGTCTCTCCCA | M7R | TGGTCTATTCATTTGACTCCACA |
| M2F | AGATGTCGCCTGATCTTGCATCG | M8F | GAATCCGAAGAGCAGCCAAC |
| M2R | GATCGACCAGGTTGCATTAACAGC | M8R | TGAAACCATGTGTCTTTTAGGGA |
| M3F | GTCGAGTAGCTTCCCTCTCT | M9F | CGGGACTATGGTATGTGGGT |
| M3R | GGTCAAGCCACTCAATCTC | M9R | TTATCTATAGTAGTGGAAAGGTGA |
| M4F | ATTAACATGACCAGTGCCTT | M10F | CGACAACGCCAAATTGATCA |
| M4R | CACTTTCAACCCTACTCAGC | M10R | TGCTAAACTTGCCGTATCTGG |
| M5F | ACTCATTTTCGGTGTCGTAG | M11F | GGGGTAATTCGTACTTTTTCATAAA |
| M5R | ACGCTTATATGGTCTGCCT | M11R | TTGCCTGATGTGCAATGCTCTA |
| M6F | ATAATTCGTGGCAACGCC |  |  |
| M6R | TCAGCGGACAGACGATAT |  |  |
