# Supplementary material for: Genetic analysis and fine mapping of a qualitative trait locus wpb1 for albino panicle branches in rice
Source: PLoS One. 2019 Sep 26;14(9):e0223228. doi: 10.1371/journal.pone.0223228 (PMC6763196; doi:10.1371/journal.pone.0223228)
Supplement: S2 Table — The list of specific primers for qPCR to examine the expression differences of the genes involved in Chl biosynthesis, photosynthesis and chloroplast development in the wpb1 mutant and NIP. (DOCX) [file pone.0223228.s006.docx]

**S2** **Table. List of qPCR primers used in this study**

| **Primer Name** | **Sequence 5'-3'** | **Primer Name** | **Sequence 5'-3'** |
| --- | --- | --- | --- |
| Actin-F | GAGTATGATGAGTCGGGTCCAG | psaA-F | GCGAGCAAATAAAACACCTTTC |
| Actin-R | ACACCAACAATCCCAAACAGAG | psaA-R | GTACCAGCTTAACGTGGGGAG |
| CAO1-F | GATCCATACCCGATCGACAT | psbA-F | CCCTCATTAGCAGATTCGTTTT |
| CAO1-R | CGAGAGACATCCGGTAGAGC | psbA-R | ATGATTGTATTCCAGGCAGAGC |
| HEMA1-F | CGCTATTTCTGATGCTATGGGT | rpoA-F | GTGGAAGTGTGTTGAATCAA |
| HEMA1-R | TCTTGGGTGATGATTGTTTGG | rpoA-R | TCTCTCTTGATCCGTAACTC |
| PORA-F | TGTACTGGAGCTGGAACAACAA | rpoB-F | TTTGGTTTCGATGTGCA |
| PORA-R | GAGCACAGCAAAATCCTAGACG | rpoB-R | TATGGTCTAATTCCGAGCGGT |
| cab1R-F | AGATGGGTTTAGTGCGACGAG | V1-F | TGGAGGTCGGGACAGAGGA |
| cab1R-R | TTTGGGATCGAGGGAGTATTT | V1-R | CGAGGAGCACCACCATCAC |
| cab2R-F | TGTTCTCCATGTTCGGCTTCT | V2-F | GAGGAGTTCCTCACGATGAT |
| cab2R-R | GCTACGGTCCCCACTTCACT | V2-R | AGCATCAATGATAGACTCC |
