# Supplementary material for: Genetic analysis and fine mapping of a qualitative trait locus wpb1 for albino panicle branches in rice
Source: PLoS One. 2019 Sep 26;14(9):e0223228. doi: 10.1371/journal.pone.0223228 (PMC6763196; doi:10.1371/journal.pone.0223228)
Supplement: S3 Table — The list of fourteen candidate genes according to BSA-Seq and ANNOVAR annotations. (DOCX) [file pone.0223228.s007.docx]

**S3 Table. Predicted genes according to BSA-Seq and ANNOVAR**

| **Gene** | **Variant** | **Chr** | **Pos** | **Ref** | **Alt** | **Annotation** |
| --- | --- | --- | --- | --- | --- | --- |
| OS01G0305900 | upstream | 1 | 11349701 | T | - | Similar to R2R3 Myb transcription factor MYB-IF35;MYB family transcription factor |
| OS01G0319400 | upstream | 1 | 12161967 | - | ATGGTGATTTTC | Protein of unknown function DUF247, plant domain containing protein;Inflorescence and seed developmental series |
| OS01G0353400  OS01G0353501 | upstream; downstream | 1 | 14166735 | - | GG | Similar to Glutathione S-transferase GST 8 |
| OS01G0342900 OS01G0343001 | upstream; downstream | 1 | 13539474 | - | CGA | Similar to Adenosine monophosphate binding protein 1 AMPBP1;AMP-binding enzyme, putative, expressed |
| OS01G0342900  OS01G0343001 | upstream; downstream | 1 | 13539511 | TAAGATAG | - | Similar to LOB domain protein 40;DUF260 domain containing protein, putative, expressed |
| OS01G0511000 | upstream | 1 | 17978206 | ACCGCGGCGCCACACCACCACATAT | - | Similar to OSIGBa0142C11.3 protein |
| OS01G0528300 | upstream | 1 | 18992647 | - | GGGG | Conserved hypothetical protein |
| OS01G0533800 | upstream | 1 | 19326817 | - | GC | Conserved hypothetical protein |
| OS01G0533800 | upstream | 1 | 19326824 | - | TGTATAT | Leucine-rich repeat domain containing protein |
| OS01G0604150 | nonsynonymous | 1 | 23788600 | T | C | Similar to mitochondrial import inner membrane translocase subunit TIM14;heat shock protein DnaJ |
| OS03G0776900 | upstream | 3 | 32208183 | C | - | Similar to R2R3 Myb transcription factor MYB-IF35;MYB family transcription factor |
