# Supplementary material for: Genetic analysis and fine mapping of a qualitative trait locus wpb1 for albino panicle branches in rice
Source: PLoS One. 2019 Sep 26;14(9):e0223228. doi: 10.1371/journal.pone.0223228 (PMC6763196; doi:10.1371/journal.pone.0223228)
Supplement: S4 Table — The list and annotations of eleven candidate genes. (DOCX) [file pone.0223228.s008.docx]

**S4 Table.** **The annotations of the candidate genes in the *wpb1* mapping interval**

| **MSU locus name** | **RAP-DB gene ID** | **CDS coordinates (5'-3')** | **description** |
| --- | --- | --- | --- |
| LOC_Os01g19894 | Os01g0304500 | 11305805- 11292665 | transposon protein, putative, unclassified, expressed; Hypothetical conserved gene. |
| LOC_Os01g19910 | None | 11307284- 11307814 | expressed protein. |
| LOC_Os01g19920 | None | 11311851- 11309445 | retrotransposon protein, putative, unclassified. |
| LOC_Os01g19930 | None | 11314011- 11312980 | retrotransposon protein, putative, unclassified. |
| LOC_Os01g19940 | Os01g0305200 | 11316601- 11320197 | expressed protein; Lg106-like family protein. |
| LOC_Os01g19950 | None | 11322752- 11321841 | expressed protein. |
| LOC_Os01g19960 | None | 11326333- 11334931 | transposon protein, putative, CACTA, En/Spm sub-class, expressed. |
| LOC_Os01g19970 | Os01g0305900 | 11350507- 11356863 | MYB family transcription factor, putative, expressed; Similar to R2R3 Myb transcription factor MYB-IF35. |
| LOC_Os01g19980 | None | 11358152- 11359942 | hypothetical protein |
| LOC_Os01g19990 | Os01g0306100 | 11365344- 11364355 | expressed protein; Plant specific eukaryotic initiation factor 4B family protein. |
| LOC_Os01g20000 | Os01g0306200 | 11367264- 11367016 | expressed protein; Protein of unknown function DUF3511 domain containing protein. |
| LOC_Os01g20020 | None | 11372793- 11374436 | hypothetical protein. |
| LOC_Os01g20010 | Os01g0306301 | 11369859- 11369593 | expressed protein. |
| LOC_Os01g20030 | Os01g0306400 | 11378988- 11379725 | expressed protein; Hypothetical conserved gene. |
| LOC_Os01g20040 | None | 11383145- 11384165 | retrotransposon protein, putative, unclassified, expressed |
| LOC_Os01g20050 | None | 11387654- 11386070 | retrotransposon protein, putative, unclassified, expressed |
| LOC_Os01g20060 | None | 11389453- 11389920 | expressed protein |
